# Electrolyte-Dependent Supercapacitor Performance on Nitrogen-Doped Porous Bio-Carbon from Gelatin

**DOI:** 10.3390/nano10020353

**Published:** 2020-02-18

**Authors:** Jie Deng, Jing Li, Shuang Song, Yanping Zhou, Luming Li

**Affiliations:** 1 College of Pharmacy and Biological Engineering, Chengdu University, Chengdu 610106, China; dengjie@cdu.edu.cn; 2 Department of Chemical Engineering, Sichuan University, Chengdu 610065, China; jingli0726@g.ucla.edu (J.L.); 2016323050027@stu.scu.edu.cn (S.S.); 3 College of Electronics and Information Engineering, Sichuan University, Chengdu 610065, China; 4 Institute of Advanced Study, Chengdu University, Chengdu 610106, China

**Keywords:** supercapacitor, electrolyte, carbon materials, ionic liquid, gelatin, high energy density

## Abstract

The carbon supercapacitance strongly relies upon the electrolyte’s nature, but the clear-cut structure–performance nexus remains elusive. Herein, a series of bio-carbons with gradually varied pore structure and surface chemistry are derived using a new salt template protocol (with eco-benign KNO_3_ as the template, activator, and porogen, and cheap gelatin as the carbon precursor), and are used as model systems to probe the dependence of the electrochemical mechanism of such nanocarbons on two typical electrolytes (KOH and EMIBF_4_). By only adjusting the KNO_3_ dosage, two pivotal figures of merit of biochar—multiscale porosity and surface functionalization—were finely modulated to construct electric double layers. Electrochemical data clarify that the combined porosity and doping effects all contribute to enhanced supercapacitance, but with only one of the two factors playing the leading role in different electrolytes. Kinetic analysis corroborates the fact that ample heteroatom doping can effectively compensate capacitance by intensive surface redox insertion in KOH, while a suitable pore size dispersion plays a preponderant part in self-amplifying the ion partitioning, and thus dictating a good charge separation in EMIBF_4_. A quasi-quantitative model of performance–structure relevance in EMIBF_4_ is judiciously conjectured to hint at a superb ion–pore-size compatibility, in which the bi- and mono-layer ion confinement coupling in integrated single and double ion-sized pores is found to be more useful for curbing notorious over-screening effects and for changing the coordination number, Coulombic ordering, and phase conformation of EMIBF_4_ in several nm-sized nanopores. This unique energy storage fashion in ion-matching pores promotes the energy density of optimal samples to a novel level of 88.3 Wh kg^−1^ at 1 kW kg^−1^, which rivals the overwhelming majority of the reported carbon materials. In short, the comparison case study here reveals a valuable correlation of carbon’s figure of merit and electrolyte type, which may act as a vital rudder to design electrolyte-contingent state-of-the-art supercapacitor materials.

## 1. Introduction

Owing to the rapid charging–discharging rate, large power density, as well as excellent longevity, supercapacitors have been widely seen as some of the most prospective energy storage devices [1,2,3,4]. Supercapacitors (SCs) by principle feature superior sustainability and are less expensive than the present battery-based initiatives because the supercapacitor systems do not depend on rare metals, noble metals, or lithium elements, but instead depend on the electrode materials and other organic or aqueous electrolytes. Supercapacitors can be categorized into electrochemical double-layer capacitors (EDLCs) and pseudocapacitors based on energy storage mechanisms. EDLCs store electricity by reversible ion electroabsorption on the electrode surfaces, while pseudocapacitors deliver energy depending upon reversible redox reactions on active materials’ surfaces. Recently, various materials (including metal oxides and hydroxides, carbon materials, conducting polymers, and hybrid composites) have been extensively studied as supercapacitor electrode materials. Of these, carbon materials are regarded as the best potential candidates for SCs, given their overarching merits of large specific surface area (SSA), rich porosity, readily modified properties (such as nanostructuring and functionalization), favorable chemical and thermal stability, environmental friendliness, low cost, precursor availability, and excellent conductivity. Since such closely concerned carbon-based SCs can conserve electricity by fast adsorption and desorption of oppositely charged electrolyte ions through electrical double layers formed around electrolyte–electrode interface regions, their electrochemical properties heavily rely on free electroactive centers facilely accessed by electrolyte ions. In this context, development of the functionalized carbon materials is in full swing [5,6,7]. However, their rather small energy densities (3–5 Wh kg^−1^) have severely limited their wide applications, particularly in comparison with the Li-ion (> 150 Wh kg^−1^) and lead acid (~30 Wh kg^−1^) batteries [8]. In theory, the energy density of EDLCs can be enhanced by heightening both the voltage window and capacitance according to the equation *E* = 0.5 *CV*^2^ [9,10]. The square dependence of the energy density on the utilized electrochemical window shows that to employ electrolytes with higher working voltage ranges and stability exerts much stronger effects on the energy density amelioration. Thus, implementing the different electrolytes, as the working fluid energy carriers for SCs cannot merely manipulate the operational voltage windows, and thus the energy level, but also permit a novel energy storage method and efficiency associated with electrochemical events occurring in several nm-sized nanopores of the functionalized electrodes. Thus, the high-voltage ionic liquid electrolytes (ILs) (such as EMIBF_4_, > 3.5 V) will feature more striking advantages over aqueous electrolytes (such as KOH, < 1.23 V), since water splitting reactions will occur in low potential ranges [11,12,13]. Disappointingly, the ILs’ supercapacitor properties are still poorly studied relative to those in the aqueous electrolytes. The behavior improvement facilely encounters a more acute challenge in that ILs possess much larger ion sizes and viscosity, and thus considerably poorer ionic conductivity and sluggish ion diffusion (especially in highly circumscribing spatial environments). For example, the ionic conductivity is only ~2 mS cm^−1^ in ILs but as high as ~1 S cm^−1^ in aqueous electrolytes [5,9]. More significantly, the susceptibility of the ILs’ electrochemical behaviors to some central factors (e.g., interaction between electrolyte ions and electrode materials [14,15], the pore size influence [16,17], and the heteroatom-doping effect [17,18]) has still defied unambiguous description, which bewilders researchers as to which properties of carbon materials should be highlighted to match with ILs. Accordingly, it is far-reaching to study the structure–performance relation depending on electrolyte and carbon electrode materials.

It has been well recognized that several essential factors, including the textural features (specific surface area (SSA), pore volume, pore width distribution), surface chemistry and heteroatom doping (B, N, O, P), and intrinsic attributes (such as the graphitization degree [19], C/O ratio), should be considered in designing high performance EDLC materials. In this regard, numerous carbon allotropes (such as the N-doped ordered mesoporous carbon, biomass-acquired porous carbon [20,21,22], and reduced graphene oxide) have shown recommendable electrochemical characteristics in aqueous electrolytes. Nevertheless, their behaviors in ILs are lacking, hindering the deep understanding of discrepancies between aqueous and IL electrolytes in supercapacitors, as well as of the paramount factors that influence the final performance. In light of the distinct natures of aqueous and IL electrolytes, the well-identified structure–performance relevance and the manipulation rationale in aqueous systems have been proven not to mechanically translate into the IL/carbon systems.

Given the perspectives above and in order to achieve more competitive energy densities, a series of nitrogen-doped porous carbons with tuned properties are devised and assessed in both KOH and EMIBF_4_ electrolytes, in the hope of offering one possible engineering criterion regarding the electrolyte type and the carbon parameter in the field of SC application. The targeted carbons can be derived simultaneously with the gradually changed nitrogen doping level and pore size distribution using a modified templating recipe with the mild KNO_3_ as both the macropore template and the nanopore porogen. Depending on the electrolyte type, such elaborated carbon materials manifest the distinct supercapacitor performance, reasons for which relate to one certain critical factor of merit of the carbon materials. Kinetic analysis clarifies that the capacitance and rate handling capability are crucially not dominated by the pore size for EMIBF_4_ but by the nitrogen dopant content for the KOH electrolyte, albeit with both effects being conducive to reinforcing electrochemical behavior. An extraordinary energy density of 88.3 Wh kg^−1^, comparable to most cutting-edge carbon materials, can be obtained for the optimally sized sample in EMIBF_4_. For EMIBF_4_, a quasi-quantitative model of the structure–performance relationship is proposed to reveal a good ion–pore-size compatibility. The model shows that integration of single and double ion-sized pores is more useful for changing the Coulombic ordering, coordination number, and phase structure of ILs in nm-sized nanopores in electrodes. This permits these SCs to possibly exceed the properties of some battery types and also ushers in an uncharted opportunity for material chemists to achieve superior energy delivery, concentrating on formulating or engineering both multiscale ion-matching pores and doping levels (rather than incessant micropores and surface areas) upon exploration of alternative electrolytes with multiple liquid conformations and compositions. Our current comparison case investigation into aqueous and IL electrolytes preliminarily sheds light on a plausible electrolyte-dependent design tenet to instruct the synthesis of exceptional carbon materials for SCs. 

## 2. Materials and Methods 

### 2.1. Materials Preparation

Four steps, including sol-gel, freeze-drying, annealing, and water washing, were involved in the synthesis of porous biocarbon. Typically, C-0.75 was synthesized as follows. Firstly, 1 g of gelatin and 0.75 g of KNO_3_ were dissolved in 20 mL of hot water at 80 °C to form a transparent sol. After cooling to room temperature, the sol was placed in a refrigerator at 4 °C for 12 h and then transferred to −20 °C for additional 12 h. Then, the frozen gel was placed in liquid nitrogen for about 30 s, followed by freeze-drying in a lyophilizer at −80 °C and 1 Pa for 36 h to obtain a light aerogel. After this, the annealing process was conducted at 800 °C for 1 h in pure Ar gas with a flow rate of 300 mL/min and a heating rate of 5 °C/min. The final product was obtained by direct water washing and drying at 60 °C for 24 h. Samples synthesized with different dosages of KNO_3_ salt (0.25, 0.5, 0.75 g) calcined at 800 °C were also obtained via the above process and designated as C-0.25, C-0.5, and C-0.75, respectively. Traces of NO_x_ emission during carbonization could be easily removed via a direct water adsorption method. All reagents were used as analytical reagents and purchased from Guoyao Chemical Co., Ltd. (Shanghai, China). All the chemicals were used as received without further treatment.

### 2.2. Materials Characterization

The morphology and structure of samples were characterized by a scanning electron microscope (SEM, JSM 7401F, JEOL Ltd., Tokyo, Japan) operated at 3.0 kV and a transmission electron microscope (TEM, JEM 2010, JEOL Ltd., Tokyo, Japan) operated at 120.0 kV. X-ray photoelectron spectroscopy (XPS) measurements were carried out on Escalab 250xi (Waltham, MA, USA). All XPS spectra were calibrated at 284.8 eV using C 1s line, and the raw data were fitted by XPSPEAK program (XPS Peak 4.1, Taiwan). The N_2_ adsorption/desorption isotherm was monitored by an Autosorb-IQ2-MP-C system (Boynton Beach, FL, USA). The specific surface area (SSA) was obtained using a multipoint Brunauer–Emmett–Teller (BET) method, and the pore size distribution (PSD) was attained via the quenched solid density function theory (QSDFT) and the equilibrium model. The X-ray diffraction (XRD) detection was implemented on a diffractometer (Bruker D8 Advance, Blerika, MA, USA) with Cu-Kα radiation at 40.0 kV and 120 mA. Raman spectra were recorded by employing a Raman spectrophotometer (Horiba Jobin Yvon LabRAM HR800, Paris, French) with He-Ne laser excitation at 633 nm.

### 2.3. Electrochemical Evaluation 

The electrode slurry was prepared by mixing 80 wt % active materials, 10 wt % acetylene black, and 10 wt % PVDF binder in N-Methyl pyrrolidone (NMP) solvent. Then, the slurry was loaded on the round-disk Ni foam (1 mm in thickness, 1.1 cm in diameter) with a mass loading of about 3 mg cm^2^. After vacuum-drying and compression under 10 MPa for 30 s, the working electrodes were attained.

Test in a 6 M KOH electrolyte: The three-electrode system was firstly evaluated in 6 M KOH, adopting the graphite as the counter electrode and the Hg/HgO as the reference electrode. The cyclic voltammetry (CV), galvanostatic charge–discharge (GCD), and electrochemical impedance spectroscopy (EIS) of all cells were appraised on an electrochemical workstation (EC Lab, Claix, France). The cyclic voltammetry (CV) and galvanostatic charge–discharge (GCD) curves were recorded in the voltage window of −1–0 V. Electrochemical impedance spectroscopy (EIS) were monitored under open circuit voltage with an amplitude of 10 mV between 10^−2^ to 10^5^ Hz. For the two-electrode configuration, two pieces of working electrodes with identical mass loading were assembled into an asymmetric capacitor. The CV and CD profiles were checked in the range of 0–1 V.

Experiment on EMIBF_4_ electrolyte: A symmetric two-electrode coin cell was constructed in pure Ar glove boxes with concentrations of both oxygen and moisture lower than the 0.1 ppm. A Whatman membrane (680 µm in thickness) made from glass microfiber (type: GF/D1823-047) was positioned between two electrode sheets with the same loading, and all of them were compressed together and sealed in a 2025-type coin cell. Electrochemical performances including the cyclic voltammetry, galvanostatic charge–discharge, and electrochemical impedance spectroscopy tests were examined. The voltage range was 0–4 V in the CV test, with the different scan rates ranging from 20 to 200 mVs^−1^. GCD detections under the various current densities of 0.5–10 Ag^−1^ were also performed between 0 and 4 V. Electrochemical impedance spectroscopy measurement was evaluated with an electrochemical analyzer within a frequency range of 10^5^–0.01 Hz.

The specific capacitance (*C*_electrode_, F g^−1^) based upon each electrode was calculated by the formula:

*C*_electrode_ = 4*I*Δ*t*/*mV* (1)

wherein *I*, Δ*t*, *m*, and *V* are the constant current (mA), discharge time (s), total mass of the active materials in the both carbon electrodes (mg), and the voltage window (V), respectively.

The energy density (*E*, Wh kg^−1^) was estimated according to the equation:

*E* = *C*_cell_*V*^2^/7.2 = *C*_electrode_*V*^2^/28.8 (2)

The power density (*P*, W kg^−1^) was obtained according to the expression:

*P* = *E*/Δ*t* (3)

## 3. Results

**Figure 1:**
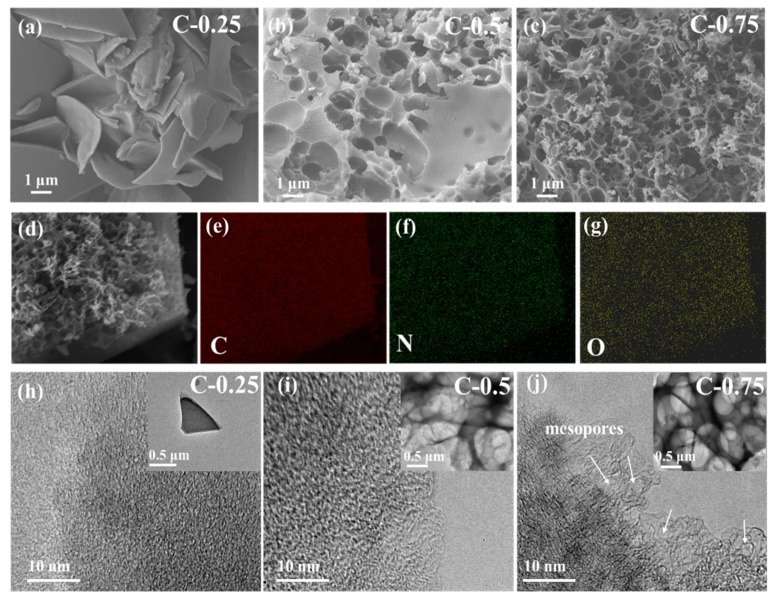
(**a**–**c**) Scanning electron microscope (SEM)images of the porous carbon. (**d**–**g**) Energy-dispersive X-ray spectroscopy EDS) mapping of C-0.75 with C, N, and O elements. (**h**–**j**) Transmission electron microscope (TEM) images.

The nitrogen-doped porous carbon was fabricated utilizing the economically effective and environmentally friendly KNO_3_ salt-template strategy based on cheap gelatin biomass. Gelatin has plentiful nitrogen and oxygen-containing groups (COOH^-^, OH^-^, NH_2_), which can directly introduce the rich heteroatoms into final carbon products [7]. To the best of our knowledge, the milder and less-corrosive KNO_3_ salt has yet to find wide applications in deriving the multifunctional carbon materials. Broadly speaking, many of the designer carbon materials in literature studies must be accomplished via a cumbersome multi-step process that combines the templates (usually NaCl, metal, metal oxide, or supermolecules), the activation agents (normally the highly corrosive strong alkaline of KOH and NaOH), and the acid leaching. In our work, KNO_3_ directly mixed with the gelatin sol in the hot water and served as both the salt-template and activation agent in the light hybrid biopolymer aerogel. In addition, the aqueously soluble and neutral KNO_3_ salt has excellent bio-compatibility with gelatin. The totally ionized species of KNO_3_ in water do not markedly change the solution’s acidity or alkalinity, totally oppressing the adverse gelatin proteolysis occurring in the non-neutral environments. Thus, this effect ensures not only the long-term survival of a uniformly complexed ion hydrogel but also its subsequent successful recrystallization into 3D salt-gelatin adducts. It needs to be pointed out that because KNO_3_ salt crystals were homogeneously confined internally into the gelatin aerogel network after freeze-drying, the efficiency of the carbonization and activation during the thermo-annealing process was greatly boosted. Finally, the residual species from the thermo-decomposition of KNO_3_ salts can be easily removed simply by water washing, contrary to the polluting acid and base leaching of traditional templates (metal, metal oxide) and activation agents (KOH and NaOH). The varied KNO_3_/gelatin mass ratio ranging between 0.25 and 0.75 (the corresponding samples are hereafter termed as C-0.25, C-0.5, and C-0.75, respectively) was used to tune the related physicochemical properties. Electron microscopy was conducted to provide microscopic insight into the materials (Figure 1**)**. Energy-dispersive X-ray spectroscopy (DES) mapping (Figure 1d–g) demonstrates the successful synthesis of N-doped carbon materials with the homogeneous N element distribution throughout the samples at a microresolution scale. The dosage of KNO_3_ salts has a great impact on the microstructure. At a lower KNO_3_ amount of 0.25, the thick platelet carbon fragments of 100–200 nm were stacked so discretely and randomly as to not assemble into the obvious frameworks with regular pores (Figure 1a,h). At higher KNO_3_ contents of 0.5 (Figure 1b and insert Figure 1i) and 0.75 (Figure 1c and inset Figure 1j), the flake structure ultimately evolved into the macroporous, interpenetrating, and interconnecting skeleton (similar to the hierarchical honeycomb or egg-box architecture), with an average macropore size smaller than 1 μm. The macropore sidewall is full of openings, clefts, or holes, revealing an exceptional spatial connectivity. These well-developed macropores can act as suitable channels to improve the mass diffusion of electrolytes during the related electrochemical processes, whereas the very discontinuous and discrete internal structures of C-0.25 render it brittle with a poor ability to resist fracture, and intensifies the transportation effects. Importantly, a close inspection shows that the numerous small-sized mesopores with graphitized and curved atom-thick sidewalls abound in C-0.75 (Figure 1j), but no visible mesopores can be observed over two other samples (Figure 1h,i). Significantly, some well-identified tiny microdomains with obvious moiré lattice fringes are embedded in the carbon skeleton in C-0.75, signifying a local graphitic structure. This graphitic structure can dramatically improve the chemical stability, electronic conductivity, and mechanic robustness. Such notable morphological difference across samples clearly show that the KNO_3_ salt dosage exerts essential impacts on the carbonization and structural evolution mechanism. 

**Figure 2:**
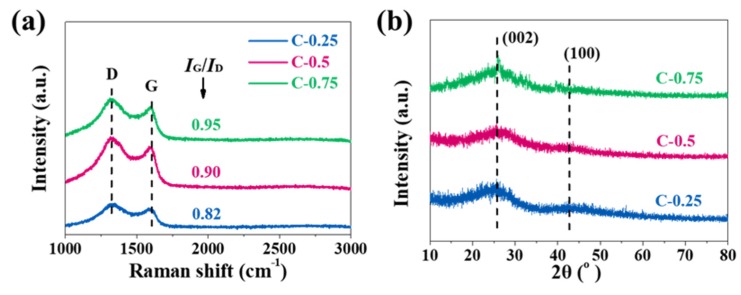
(**a**) Raman and (**b**) the X-ray diffraction (XRD) patterns of all porous carbons.

The crystallographic structure and degree of graphitization of carbon materials were determined by Raman spectra, which is widely deemed as a robust technique to characterize the structure of graphitic carbon materials. Generally, the Raman spectrum of a graphitic carbon material comprises the typical D- and G- bands. The former (1330–1340 cm^−1^) can relate to the defect or disorder-activated breathing modes of six-membered carbon rings, and the latter (1580–1600 cm^−1^) typifies the E_2g_ phonons at the Brillouin zone center [23]. For all samples, the representative G band at ~1580 cm^−1^ and D band at ~1320 cm^−1^ are well identified (Figure 2a). Crystallinity, as quantified by an intensity ratio of the G to D bands (*I*_G_/*I*_D_), is 0.82, 0.90, and 0.95 for C-0.25, C-0.5, and C-0.75, respectively, manifesting a gradual gain in the graphitic degree with the higher KNO_3_ dosage and the gradual reduction in the defect density in carbon. The incremental *I_G_/I_D_* elucidates that in our method the augmented KNO_3_ dosage seems to be non-destructive but conductive to the graphitic structure formation. The enhanced graphitization may come from the fact that plenty of the heat emissions due to fierce carbon reduction reactions may generate a high-temperature zone localized at peripheries of the newly-minted pores to consolidate the aromatization and exfoliation of the bulk carbon layers by the in-situ-formed K species (during the carbon reduction). The crystallinity and phases of samples were further verified by X-ray diffraction (XRD) measurement (Figure 2b). The patterns show two diffraction peaks attributed to (002) and (100) planes of graphite (JCPDS No. 41-1487) [7]. Evidently, the broadening characteristic of peaks confirms a disordered structure of the as-obtained porous carbon materials. Combined with Raman and transmission electron microscope (TEM) data, it can be deduced that the N-doped porous biochar, particularly C-0.75, features a long-ranged amorphous but short-ranged ordered structure.

**Figure 3:**
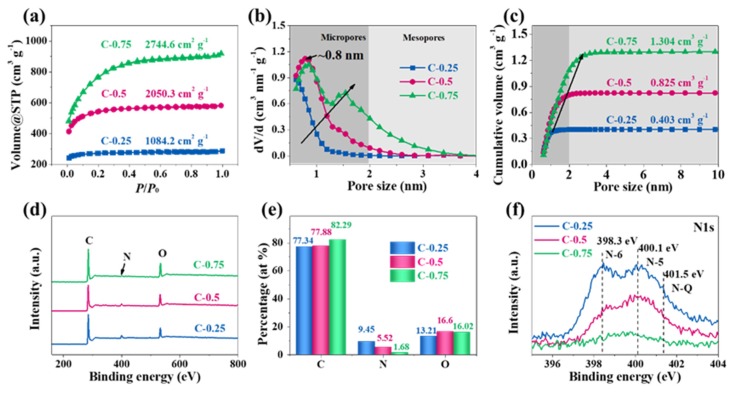
Characterizations of the surface chemical properties and porous texture structures. (**a**) N_2_ adsorption-desorption isotherm curves, (**b**) pore size distribution, and (**c**) the cumulative pore volume. (**d**) The full-scale X-ray photoelectron spectroscopy (XPS) pectra. (**e**) Bar chart of the content variation of C, N, and O elements with the change of KNO_3_/gelatin ratios. (**f**) High resolution N1s spectra.

N_2_ adsorption experiments were conducted to investigate the texture properties of each material. In Figure 3a, the adsorption–desorption isotherms present I-type curves without hysteresis, and the adsorption branch compactly superposes the desorption branch, meaning the micropores dominate the materials. The specific surface area (SSA), determined using multi-point the Brunner–Emmett–Teller (BET) method, amounts to 1084.2, 2050.3, and 2744.6 m^2^ g^−1^ for C-0.25, C-0.5, and C-0.75, respectively. Notably, all carbon products display narrow pore size distribution (PSD) but distinct variation (Figure 3b). The pores in C-0.25 are located at the micropore zone in the range of 0.5–1 nm and are predominately centered at 0.5 nm. The SSA and pore volumes contributed by mesopores (defined as *S*_meso_ and *V*_meso_) are only 45.5 m^2^ g^−1^ and 0.001 cm^3^ g^−1^ (Table 1). These data indicate that C-0.25, in reality, can be viewed as a totally ultra-microporous material. In the case of C-0.5, the PSD positively shifts to the region of 0.5–2 nm, the overwhelming majority of which are centered around 0.8 nm. This clearly shows that the larger KNO_3_ salt quantity can enlarge the micropore size up to 0.8 nm. The pore size increase is due to the expanding effect caused by more pyrolytic gases during the carbon reduction reactions. Meanwhile, the SSA and pore volume originating from micropores can reach 1770.1 m^2^ g^−1^ and 0.718 cm^3^ g^−1^, respectively. As for C-0.75, the pore size further increases while the proportion of the specific mesopore surface area and volume equals 32.1% and 36.8%, respectively. This coincides with the well-observed mesopores in TEM images (Figure 1j), suggesting a large volume contribution from small-sized mesopores (2–3 nm). This phenomenon totally differs from the picture that the micropores only contribute to the pore volume of C-0.25 and C-0.5. In conjunction with PSD, it can be inferred that the C-0.75 practically has a dual-mode pore configuration, with predominant pore widths of 0.8 and 1.5 nm. 

The surface chemical state and elemental compositions were analyzed by X-ray photoelectron spectroscopy (XPS). The survey spectra (Figure 3d) show that C, N, and O elements exist in each sample. The atomic percentage shows that C-0.25 possesses the highest heteroatom doping level (i.e., a nitrogen fraction of 9.45%), in sharp contrast with a minimal N content of 1.68% over C-0.75 (Figure 3e). The downward dopant evolution trend reveals the fact that the upward KNO_3_ dosage is detrimental to the N-doping. In fact, the C-bonding and N-C locations also change with the KNO_3_ dosage. The high-resolution N1s spectra (Figure 3f) can be further deconvoluted into pyridinic N (N-6, 398.3 eV), pyrrolic N (N-5, 400.1 eV), and graphitic N (N-Q, 401.5 eV) [24,25,26], and the much stronger peak intensity of C-0.25 also exemplifies its higher N-doping level. As the KNO_3_ dosage increases from 0.25 to 0.75, the N replacements around the “regular” graphitic positions (N-Q) rather than defective situations (N-5 and N-6) gradually increase, accordant with the *I_G_/I_D_* ratio evolution in the Raman spectra. In other words, the higher KNO_3_ dosage can make the less steady N-5 and N-6 configurations appreciably transform without diminishing the relative percentage of the steadier N-Q structures. The N-C bonding and location evolution within the carbon framework most possibly correlates with the activation etching process. This process initiates from the chemically more active nitrogen defect sites and propagates in base planes of carbon lattices to create the increasingly greater pores with the rising KNO_3_ dosage, as clearly shown by TEM and Raman studies in Figure 1 and Figure 2. All taken together, the comparison data doubtlessly expound that C-0.25 was not fully activated by KNO_3_ during pyrolysis, thus inducing the undeveloped pores and framework, inappreciable mesopores, and low SSA; surprisingly, it can still preserve the richer N element from raw materials than C-0.5 and C-0.75. As universally acknowledged, texture properties of both high SSA and appropriate pore breadth are in great favor of reinforcing the EDLC capacitance, while the heteroatom doping is capable of further boosting the performance by Faradic reactions or pseudocapacitance. However, within this report, we have found that the effect of these two factors upon capacitance is strongly influenced by the used electrolytes.

**Figure 4:**
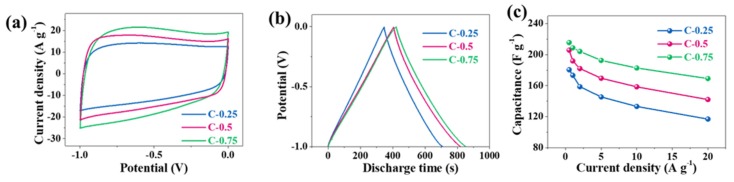
Three-electrode performances in 6 M KOH aqueous electrolytes. (**a**) The cyclic voltammetry (CV), (**b**) galvanostatic charge–discharge (GCD), and (**c**) rate capability.

**Figure 5:**
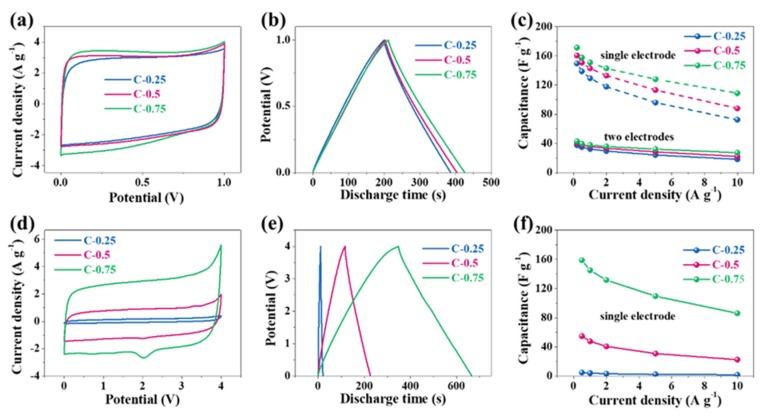
Electrochemical performance of the two-electrode symmetric supercapacitor in (**a**–**c**) 6 M KOH electrolyte and (**d**–**f**) EMIBF_4_ ionic liquid electrolytes (ILs). (**a**,**d**) Cyclic voltammetry (CV) curves conducted at 20 mV s^−1^, (**b**,**e**) galvanostatic charge–discharge (GCD) curves conducted at 0.5 A g^−1^, and (**c**,**f**) rate capability ranging from 0.5 to 10 A g^−1^ for three samples with various pore size distribution (PSDs) and doping levels.

The electrochemical performance was evaluated in two typical electrolytes, 6 M KOH and EMIBF_4_ ILs, which have operational voltage windows of 1 V and 4 V, respectively. We firstly assessed the three-electrode performance in 6 M KOH (Figure 4). All CV curves in aqueous solution deviate from the standard rectangle due to the shape distortion (the wide bulge at the low potentials), suggesting the departure from the ideal EDLC behavior. Based on a single electrode, the area of the CV curve for C-0.75 slightly outnumbers both C-0.25 and C-0.5. The capacitances at 0.5, 1, 2, 5, 10, and 20 A g^−1^ are calculated in sequence to be 215.6, 208.8, 204.2, 192.6, 182.8, and 169.2 F g^−1^ for C-0.75; 205.8, 191.8, 182.1, 169.6, 158.5, and 142.1 F g^−1^ for C-0.5; and 180.5, 173.4, 158.7, 145.4, 133.2, and 116.9 F g^−1^ for C-0.25. The corresponding electrical capacity retention is 78.5% for C-0.75, 69.0% for C-0.5, and 64.8% for C-0.25. The fading rate capability might closely correlate with the falling pore size. It should be stressed that the capacitance gap among these samples is quite nuanced. For instance, the capacitance of C-0.75 is only 35 F g^−1^ greater than that of C-0.25 at 0.5 A g^−1^, whereas the SSA and pore volume of C-0.75 are ~2.5- and ~2.1-fold larger than those of C-0.25, respectively. When solely considering the micropore’s contribution to constructing an effective EDLC structure, the *S*_micro_ of C-0.75 is also ~1.8 times as high as that of C-0.25, which, in theory, should at least have resulted in a roughly 1.8-fold capacitance boost instead of the experimentally similar values. Therefore, the striking difference in textural properties (SSAs and pore volumes) does not underlie the subtle electrical capacity variation at all. More importantly, such delicate disparity will be further bridged in two-electrode evaluation systems because the electrical capacity, theoretically, would be a quarter of that in the three-electrode system. This could be further demonstrated by the following data tested in a two-electrode system. It can be seen that CV curves (Figure 5a) partially superpose each other and the CD curves (Figure 5b) nearly overlap mutually, hinting at a very analogous electrical capacity among the three samples. The quantified electrical capacity based on a two-electrode configuration approaches around 40 F g^−1^ for all electrodes, finally giving a single-electrode value (dash lines) of ~150–170 F g^−1^ at 0.5 A g^−1^ (Figure 5c). Thus, the big contradiction between the similar electrical capacity and the distinct textural parameters can only be interpreted in view of a huge discrepancy in the heteroatom doping. In addition to EDLC, the high doping level of C-0.25 might contribute to extra electrical capacity and fill the gaps relative to the C-0.5 and C-0.75 electrodes with high SSA and pore volumes. On the other hand, the foregoing studies show that the three-electrode electrical capacity of a series of N-doped biochar defies direct translation into electrochemical cells. As a matter of fact, the carbon-based substances are peculiar because they can function as either negative electrodes or positive electrodes in the symmetrical electrochemical systems, with a theoretical per-electrode specific electrical capacity almost identical to the counterparts assessed by the three-electrode analysis. On the contrary, as widely reported in literature, the pseudocapacitive substances possessing palpable bumps (normally redox signals) can generally backfire for symmetrical supercapacitor systems because their two differential electric capacities at cathodes and anodes, referred to as C_1_ and C_2_, become unequal [27,28]. In a common case, if one carbon electrode has a proper voltage towards the main bumps of CV curves, and therefore a greater differential electric capacity, the other carbon electrode must encounter a voltage far away from these main bumps, thus triggering a much lower differential electric capacity. Since a symmetric supercapacitor is seen as electronically equivalent to the two serial capacitors, their overall electric capacity of C_1_C_2_/(C_1_ + C_2_) cannot exceed the largest theoretical value, which can amount to 1/2C_1_ = 1/2C_2_ on the condition of C_1_ = C_2_. There must stand no chance of satiating the optimal electrical capacity under every voltage, unless CV shapes must remain perfectly symmetrical. In effect, due to the notable contribution by redox reactions, the N-doped porous carbon electrodes exhibit somewhat distorted CV curves (Figure 4a). In other words, a variable differential electrical capacity takes place during the three-electrode evaluation. As a consequence, their two-electrode system is projected to be unable to meet C_1_ = C_2_, thereby triggering a symmetrical electrical capacity different from the one derived from the three-electrode analysis. The two-electrode CV patterns (Figure 5a) also keep the misshaped orthogons with the low regularity and give rise to a per-electrode electrical capacity of 150–170 F g^−1^ at 0.5 A g^−1^, in the range of 16.9–17.4% loss of the three-electrode electrical capacity of 180.5–215.6 F g^−1^. To put it another way, all N-doped carbon electrodes, each featuring obvious deformation within CV profiles, encounter an apparent electrical capacity degradation while applied in two-electrode tests. All in all, the notably varied differential electrical capacity, as revealed by the apparent inconsistency or inequality between two-electrode and three-electrode assessments, indeed substantiates the existence of the marked pseudo-capacitance in the N-doped porous carbons in the KOH aqueous solution.

We also tested the electrochemical performance of N-doped porous carbons in the EMIBF_4_ IL electrolyte. Interestingly, in sharp contrast with the results in KOH, the CV area of C-0.25 (Figure 5d) is dramatically lower than C-0.5 and C-0.75, along with the CD curves (Figure 5e). Their CD electrical capacity based on a single electrode at a current density of 0.5 A g^−1^ is as high as 158.9 F g^−1^ for C-0.75, in contrast to the smaller values of 54.7 F g^−1^ for C-0.5 and 4.8 F g^−1^ for C-0.25, a startling enhancement of three times and more than one order of magnitude, respectively (Figure 5f). The electrical capacity retention can reach 1.7 (35.0%), 22.4 (41.0%), and 85.9 F g^−1^ (54%) at 10 A g^−1^ over C-0.25, C-0.5, and C-0.75, respectively, suggesting dramatically promoted rate handling with a higher KNO_3_ dosage. These data clearly uncover that C-0.25 is almost unable to store energy, while C-0.75 can deliver a high electrical capacity, even comparable to the most advanced carbon-based materials in IL electrolytes. Intriguingly, the capacitive evolution with the KNO_3_ dosage in EMIBF_4_ seems quite dissimilar to the variation trend in KOH, which in nature shows that the decisive factor or the energy storage mode is distinct in the two electrolytes. Given that the sequence of the electrical capacity improvement (from 4.8 F g^−1^ for C-0.25 to 54.7 F g^−1^ for C-0.5 and 158.9 F g^−1^ for C-0.75) is in accordance with the order of the doping level dropping from 9.45% for C-0.25 to 5.52% for C-0.5 and to 1.68% for C-0.75, the great electrical capacity promotion from C-0.25 to C-0.75 could not be explained by heteroatom doping as the leading factor (as analyzed previously in KOH electrolytes, because the N/O doping in C-0.75 is the lowest). In this case, rather than the doping, the textural properties, specifically the pore size, have the decisive effect on tuning the capacitance in the EMIBF_4_ IL electrolyte, which is opposite to that in the KOH electrolyte. 

**Figure 6:**
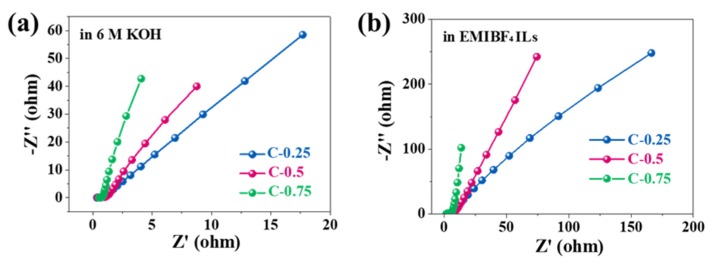
Electrochemical impedance spectroscopy (EIS) spectra of the samples tested in (**a**) 6 M KOH and (**b**) EMIBF_4_ ILs.

**Figure 7:**
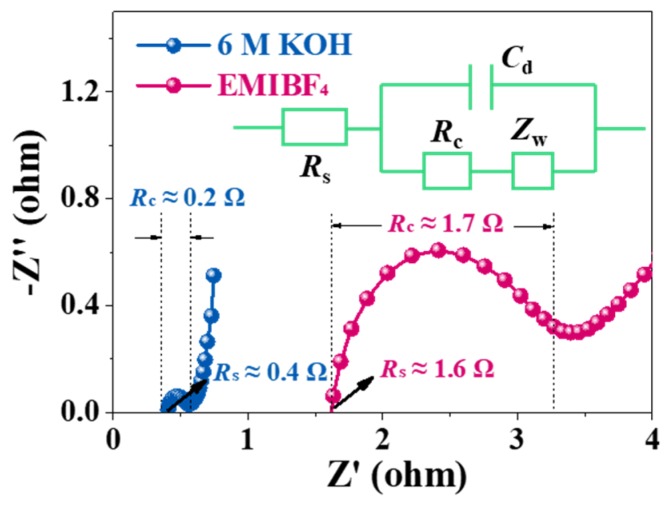
The comparison of the EIS spectra of C-0.75 in 6 KOH and EMIBF_4_ ILs.

Nevertheless, the mass diffusion and rate capability were greatly influenced by the pore size, regardless of using KOH or EMIBF_4_. To probe the impact of textural traits on the transport kinetics, electrochemical impedance spectroscopy (EIS) (Figure 6) was performed on all samples. All Nyquist plots show a half-cycle in high frequencies, a 45^°^ slope in the middle frequency segment between 5 and 100 Hz, and near-straight lines under frequencies smaller than 1 Hz. Such characteristics imply the typical capacitive processes of carbon-based materials. The capacitive processes can be further corroborated analytically through an equivalent circuit for porous electrodes (insert in Figure 7). The projection of the 45^°^ inclined lines onto abscissa denotes the ionic resistance for electrolytes (filled in pores of porous electrode structures), which can serve as a central pointer to indicate an ion diffusion process and the rate handling capability of porous N-doped biochar electrodes during charging and discharging. Irrespective of electrolyte type, the gradual diminishment in the projected length values from C-0.25 to C-0.75 shows a drop in the ionic resistance. This dropping trend consists of the pore width increase in the porous N-doped biochar skeleton. The direct lines at low frequencies are nearly vertical for C-0.75 but are rather skewed for both C-0.5 and particularly C-0.25, revealing much faster ion diffusion with increasing pore size. As for the diffusively optimized porous architecture (C-0.75), the ionic resistance (*R*_s_) extrapolated from the *x*-intercept is only ~0.4 Ω in KOH electrolyte, one magnitude smaller than that in EMIBF_4_ (~1.6 Ω). The charge transfer resistance (*R*_c_) (implied by a depressed high-frequency semi-circle) is also as small as ~0.2 Ω in KOH, in contrast to ~1.7 Ω in EMIBF_4_. These distinct values must result from the notable differences in the ion dimension and viscosity, thus essentially reflecting the varying transport dynamics of different electrolyte ions in the same pore structures.

In order to explore the in-depth reasons for electrochemical differences in KOH and EMIBF_4_, the capacitance contribution by the reversible capacitive behavior or the diffusion-controlled insertion (Faradic reactions) behavior is studied from the relationship between the current (*i*) and the scan rate (*v*) [29,30,31]

*i* = *av^b^* (4)

This equation shows that the electrochemical process is diffusion-controlled (or Faradic-inserted) when b = 0.5 (it can describe both the ion transfer process and the redox reaction), or a rapid capacitive process when b = 1. 

Equation (1) can also be expressed by another form:

*i*(V) = *k_1_v* + *k_2_v^1/2^* (5)

where *k_1_v* represents the capacitive effect current and *k_2_v^1/2^* is the diffusion-controlled current, expressed by:

*i*(V)/*v^1/2^* = *k_1_v^1/2^* + *k_2_* (6)

**Figure 8:**
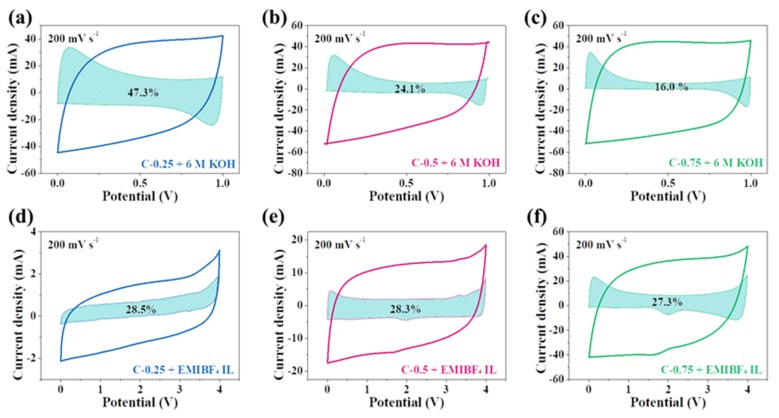
CV curves of (**a**,**d**) C-0.25, (**b**,**e**) C-0.5, and (**c**,**f**) C-0.75 in two kinds of electrolytes (6 M KOH and EMIBF_4_ IL) tested in a two-electrode system at 200 mV s^−1^. The shadow areas represent the diffusion-controlled contribution.

In this step, linear fitting of voltammetric currents at each potential can be implemented, and the coefficients *k_1_* and *k_2_* can also be determined to calculate the *k_1_v* and *k_2_v^1/2^*. Then, the proportion of the diffusion-controlled current or capacitive current can be determined. The fitting results at a current density of 200 mV s^−1^ are presented in Figure 8. The shadow areas represent the diffusion-controlled process, which reflected the pseudocapacitance from the heteroatom doping via interfacial redox reaction. Remarkably, the pseudocapacitance contribution in 6 M KOH is 47.3% for C-0.25, but24.1% in C-0.5 and 16.0% for C-0.75. However, the overall CV area of these three samples nearly equals each other, further confirming the important role of doping in enhancing the total capacitance. The proportion of the pseudocapacitance is in good accordance with the corresponding doping level, namely the larger the dopant, the greater the pseudocapacitance. This unambiguously casts light upon why C-0.25, having much smaller SSA and pore volume, could deliver a comparable capacitance to C-0.75, which has much greater SSA and pore volume (215.6 vs. 180.5 F g^−1^ at 0.5 A g^−1^). This significant discovery can serve as an engineering norm to guide the rational design of carbon materials for SCs in aqueous systems; that is, instead of the endless pursuit of textural optimization, particularly including the prodigious specific surface and highly hierarchical porosity, the appropriate nitrogen dopant content can more effectively boost the supercapacitive behavior. This is mainly because the practical applications of the highly hierarchical porous carbons with large surface areas have always been restrained by their small tap density (which is very detrimental to the volumetric performance) as a result of the existence of empty space and other topological problems. Worse still, securing the empty spaces deprives the materials of vital factors during the tight calendaring because the majority of empty voids may undergo the utter devastation. These considerations can further stress the superiority of proper doping to high porosity for supercapacitors (SCs) in aqueous systems. 

Interestingly, in EMIBF_4_, the diffusion-controlled process for all carbon electrodes accounts for ~28%. In spite of the huge difference in the nitrogen dopant content across samples (9.45% for C-0.25, 5.52% for C-0.5, and 1.68% for C-0.75), there is only a trivial change concerning the contribution from redox reactions. This doping-independent or -insensitive phenomenon here shows that the nitrogen doping is not a critical factor in heightening the carbon capacitance in the high-voltage ionic liquids. Results also confirm that the energy storage events must largely depend upon adsorption and desorption instead of the insertion or redox reaction relevant to the nitrogen dopant when using EMIBF_4_ as an electrolyte. This is primarily because KOH, as a type of protic electrolyte, in water can offer a good reaction media for redox reactions of heteroatoms. Contrastingly, because EMIBF_4_ is a pure molten salt with a great ion size and steric hindrance capability, as well as good electrochemical stability, under its theoretical operation voltage, it is very tough for it to intensively interact with functional groups on carbon surfaces. Therefore, the rich doping of C-0.25 does not trigger a notable improvement in the capacitance, such as it does in KOH. In fact, the nitrogen dopant most possibly calls into play the good wetting of carbon surfaces by EMIBF_4_ to augment the ion-philicity. Of course, the precise roles of N-dopants in modifying the supercapacitive behavior of EMIBF_4_ on carbon materials requires investigation via a coordinated combination of cutting-edge in situ or in operando characterization technologies, which is out of the scope of our studies here. For the sake of clarity and succinctness, Table 2 shows all central metrics for all applied samples in both electrolytes.

**Figure 9:**
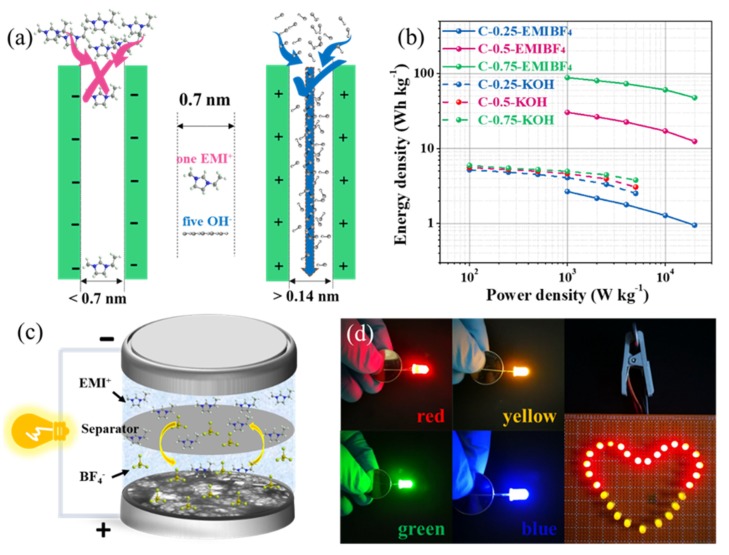
(**a**) Proposed pore-dependent ion diffusion models using two kinds of electrolytes (KOH and EMIBF_4_) with different ion sizes. (**b**) The Ragone plot shows the energy–power density relationship of the two-electrode symmetric capacitor used in different electrolytes. (**c**) Illustration of the assembled 4 V coin cell using C-0.75 as the electrode material, and (**d**) its practical demonstration in lighting LEDs with different colors and working voltages, or in parallel.

Having safely ruled out the doping effect, the pore size is a decisive factor in this case. Given that the pore size of C-0.25 is mainly centered at 0.5 nm (even smaller than the diameter of the desolvated EMI^+^ ions (~0.7 nm) [16]), the abundant ultra-micropores were inaccessible to the EMIBF_4_ ILs and the microporous interfaces could not be effectively utilized (Figure 9a). However, in KOH, the abundant ultra-micropores allow the OH^-^ ions to get through channels owing to the much smaller size of the bare OH^-^ (~0.14 nm) (note that we only take into consideration the bare size of the electrolyte ions because the solvated ions would firstly be desolvated if their diameter was larger than that of the pores). That is to say, the minimum size of the pores that can construct the double electric layer to store energy is about 0.14 nm for the KOH electrolyte but is 5 times larger for the EMIBF_4_ electrolyte. Therefore, the critical pore size to deliver energy is at least 0.7 nm for EMIBF_4_. In this case, pores in C-0.25 were “alive” for OH^-^ but “dead” for EMI^+^. As a result, the larger-sized (0.8 nm) micropores coupled with the partial double-ion size sub-mesopores (1.5 nm) in C-0.75 can largely enhance the ion-pore size compatibility to result in the significantly promoted capacitance in EMIBF_4_.

Briefly, we compared two common electrolytes, KOH and EMIBF_4_ ILs, and their influences upon the EDLC performance on N-doped porous carbon materials with tunable texture properties and surface doping chemistry. On account of the smaller ion size of the KOH electrolyte, ultrasmall micropores were still available for C-0.25. Additionally, the extra pseudocapacitance from redox reactions of rich heterodoping in C-0.25 further offsets the capacitance gaps compared with C-0.75 and C-0.5, which have relatively higher SSAs and pore volumes. However, the smaller pore size causes a very sluggish ion diffusion and decreased rate capability in both electrolytes. For EMIBF_4_, doping is not the determining prerequisite, while the pore size dispersity has a greater impact on the carbon capacitance performance. Although C-0.75 has the highest capacitance in both KOH and EMIBF_4_, the results tell us that doping is viewed as a more fruitful protocol to reinforce performance in aqueous electrolytes, while pursuing a suitable pore size is counted as more rewarding than doping for IL electrolytes. This provides a definite direction to reasonably prepare the electrolyte-dependent carbon materials for SCs.

Energy and power densities are given in the Ragone plot (Figure 9b). Most significantly, the Ragone plot shows a rather parallel line plot to the x-coordinate when increasing from 1 kW kg^−1^ up to a very high specific power density of 20 kW kg^−1^, indicative of a superior energy–power density rating. Specifically speaking, an energy density as high as 88.3 Wh kg^−1^ is obtained for C-0.75, 30.4 Wh kg^−1^ for C-0.5, and only 2.7 Wh kg^−1^ for C-0.25 at 1 kW kg^−1^. An extraordinary energy density of 47.7 Wh kg^−1^ for C-0.75 is still kept at 20 kW kg^−1^, which substantially surpasses those of the other two samples and the values in KOH (5.2 Wh kg^−1^ at 0.1 kW kg^−1^ and 2.5 Wh kg^−1^ at 5 kW kg^−1^), revealing a fascinating energy retention trait. The energy–power density rating performance of our optimal sample can rival the overwhelming majority of the reported advanced carbon materials, as compared in Table 3. [12,19,21,32,33,34,35,36,37,38,39,40,41,42,43,44,45,46,47,48,49,50,51,52] Such outcomes not only advance the facile or efficient concept of maturing the heteroatom-doped porous nanocarbons achieved from the biomass, but also overcome a disharmonious energy and power density clash via coordinating the well-engineered carbon electrodes with the electrolytes that feature a broad working potential range. To further demonstrate the practically relevant applications of the materials, a 4 V two-electrode coin-cell was configurated, as illustrated in Figure 9c. It can light LEDs with different colors (red, yellow, green, and blue) between the working voltages of 1.8–3.5 V (Figure 9d). In addition, one coin cell can directly power 27 LEDs in parallel with a heart-shaped pattern (Figure 9d). This example heralds an appealing prospect for real-world usage.

**Figure 10:**
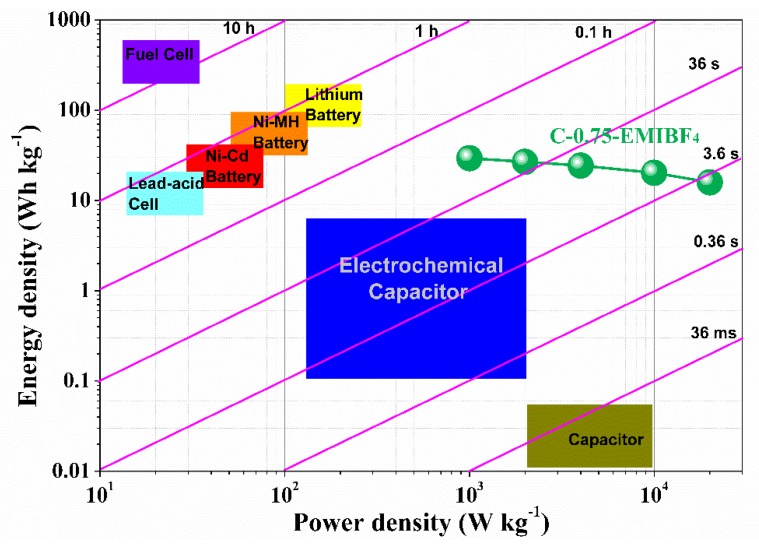
Comparison of the C-0.75-based packaged supercapacitor in EMIBF_4_ with other commercial electrical energy storage devices (data adapted from [54]).

Given that in addition to the carbon-based electrodes, the overall mass of an actual supercapacitor device comprises some inactive and passive components, such as current collectors, separators, binders, and packaging, the Ragone plots built only on the mass of the electroactive material are unable to provide a pragmatic assessment of the energy and power performance. Because the carbon weight accounts for roughly 30% of the commercial packaged supercapacitors (typical for large-scale AC-based supercapacitors), a factor of between 3 and 4 is universally adopted to extrapolate the real-world energy and power values from the behavior of the electroactive substances in a packaged cell [53]. Figure 10 shows both Ragone plots of a C-0.75-based packaged supercapacitor in EMIBF_4_ and a detailed contrast to other commercial electrochemical energy storage setups. The practical specific energy density of the postulated packaged SCs can expectedly reach a high value of approximately 15.91–29.43 Wh kg^−1^, significantly surpassing the commercial carbon-based supercapacitors (3~5 Wh kg^−1^), two times higher than that reported for carbon oxide hybrid electrochemical devices, and even escalating into ranges offered by batteries. Particularly, such normalized energy densities can strongly compete with the lead-acid batteries and nickel metahydride (Ni-MH) batteries (Figure 10). Normalized power densities are 0.33–6.67 kW kg^−1^, which outnumbers both those of Ni-MH batteries by one order of magnitude (Figure 10), while the values from commercial carbon supercapacitors have energy density values of 4 to 5 Wh kg^−1^. Therefore, our devices are on par with those commercial energy storage devices. More importantly, the “representative time” likewise discloses that the setup can accomplish a charging–discharging cycle within just a few seconds under maximized power handling of 20 kW kg^−1^ (Figure 10). The foregoing results explicitly signify that a C-0.75-based packaged supercapacitor in EMIBF_4_ possesses immense potential in future realistic adhibitions, which could bridge the performance–material gulf between supercapacitors and batteries.

## 4. Discussion

In fact, the supercapacitive behavior of EMIBF_4_ on a series of C-0.25, C-0.5, and C-0.75 is interesting and thought-provoking. Such impressive capacitive function must initiate from optimal pore structure effects in terms of the mixed micro-/mesopores and the occurrence of unique electrochemical processes. This particular electrochemical event must arise within volumes of ILs in place of the surface EDL condensation, as will be concisely discussed below. Surprisingly, the purely ultra-microporous C-0.25 owns empirically recommendable textural parameters with both a sizable specific surface area (1084.3 m^2^ g^−1^) and pore volume (0.403 cm^3^ g^−1^), but it is nearly inactive in terms of storing energy (4.8 F g^−1^ at 0.5 A g^−1^). In sharp contrast, an 2–2.5 times increase in the specific surface area (2744.6 m^2^ g^−1^) and pore volume (1.304 cm^3^ g^−1^) can incredibly lead to a 33-fold capacitance boost (158.9 F g^−1^). This anomaly seems very compelling but counterintuitive, severely deviating from the manner in which the capacitance typically behaves for well-identified aqueous electrolytes. This doubtlessly shows that the tailored porosity (rather than the “incessantly” sought specific surface area and heteroatom functionalization) serves as an underlying prerequisite to sustain the efficient charge storage in IL-soaked carbon SCs through a special working mechanism. In fact, the pore width of purely ultra-microporous C-0.25 is dominated at about 0.5 nm, and is much smaller than the crystallographic diameter of desolvated EMI^+^ ions (~0.7 nm). Thus, its rich ultra-micropores are too narrow for EMIBF_4_ to sample to build up abundant EDLs, even during a slow discharge and charge stage at 0.5 A g^−1^. Because of the charge inversion, ion species from ILs have to adsorb and periodically reorientate in the shape of a long-range ordered multilayer (layer-by-layer) assembly of alternately distributed cations and anions outside the charged ultra-microporous surfaces of C-0.25. The oppositely charged ions in this layout almost adopted the same Coulomb ordering structure as the IL bulk phase, and thus had sufficient high long-range mutuality to conjugate the polarization of the headmost adsorption layer with that of a contiguous layer [55,56]. This results in the damping charge density oscillation, which means that the first counter-ion tier can achieve a peak density while the next co-ion-rich layer has a smaller density (still in excess of bulk values). That is, to satisfy the charge neutrality for the entire system, the charges of the first adsorption layer outnumber those of the electrode surfaces but are immediately offset by the second layer. This compensation behavior can propagate beyond a few tiers, such that a tiny portion of absorbates are efficiently utilized during electricity input and output. Finally, such harmful lattice saturation and overscreening effects occur, leading C-0.25, which has large surface areas of 1084.3 m^2^ g^−1^, to only exchange a rather small amount of energy of 4.8 F g^−1^.

Because pores in C-0.5 are 0.8 nm in diameter (nearly identical to the size of naked EMI^+^ ions), they can exclusively accommodate a single layer of electrosorbed ions. These electrosorbed ions can be driven into micropores through a good combination of external potentials, capillary forces, ion–wall electrostatic interactions, image forces, and so on. Then, they will undergo all kinds of atomic-scale local microenvironments inside ion-size pores to break the alternating Coulomb array patterns of anions and cations, with both an appreciable co-ion paring generated in the nearest coordination shells around a central ion and image counter charges arising in sidewall carbon atoms. In this case, all of the charges conveyed by the first adsorption tier in pores can more precisely counterpoise the images of the carbon pore sidewalls to contribute to a far superior efficacy; the affinity of the ions in the first layer to the sidewall surfaces is not neutralized by the well-assembled abutting co-ion layer, thereby self-amplifying the ion partitioning and surface charging. Such distinct in-pore ion arrangement must suppress the so-called multilayer structures that arise in C-0.25, changing the ion partitioning into the ion-sized micropores and the interfacial properties of the ionic species [57,58]. As a result, the capacitance in the ion-sized pores of C-0.5 can increase to 54.7 F g^−1^; namely, only a 1.9-fold increase in specific surface areas surprisingly brings about one order of magnitude gain in capacitance relative to C-0.25. For C-0.5, its total specific surface area is 2050.3 m^2^ g^−1^, with a microporous contribution of up to 1770.1 m^2^ g^−1^. The total and microporous volumes equal 0.825 and 0.718 cm^3^ g^−1^_,_ respectively. Considering a very narrow pore size distribution, the surface area and pore volumes must be mainly determined by the ion-sized pores (measuring about 0.8 nm). Thus, the obvious capacitive enhancement must have a close relation to the unique ion partitioning under the single-layer confinement.

Intriguingly, apart from the ion-sized micropores (0.8 nm), a certain fraction of double ion-sized pores (1.5 nm) are successfully created in C-0.75. More importantly, the total surface area and pore volumes reach 2744 m^2^ g^−1^ and 1.304 cm^3^ g^−1^, respectively; the microporous surface areas and volumes still maintain values of 1864.1 m^2^ g^−1^ and 0.826 cm^3^ g^−1^, very similar to the C-0.5 counterparts. According to the foregoing charge storage mechanisms of C-0.5, a 1.3-fold increase in the specific surface areas of C-0.75 relative to C-0.5 should give a capacitance of about 92 F g^−1^ rather than the dramatic growth to the anomalous experimental value of 158.9 F g^−1^. More specifically, a comparable capacitance between C-0.5 and C-0.75 should have been achieved because they both have nearly the same micropore surface areas and volumes. Quantitatively, a surface area of 880.5 m^2^ g^−1^ from mesopores (32.1%) seems to contribute to 42.1% of the overall capacitance. This phenomenon apparently clashes with a previous viewpoint on the ion sieving effect, which suggests that only when an ion perfectly adjusts itself to pore sizes to minimize the free spaces does the maximal capacitance of ILs inside the nanopores occur at the critical pore/ion size ratio of ≈ 1 [59,60]. On the grounds of such perspectives, the unique ion partitioning mechanism for C-0.5 should have been obscured for C-0.75, which has a much wider PSD ranging from 0.8 to 1.5 nm, because the experimentally measured capacitance could be averaged over a wide pore width. This obscuring or averaging effect should have greatly lowered the total capacitance on C-0.75 instead of it amazing increasing to 158.9 F g^−1^. Actually, the mono- and bilayer confinement effects co-exist and robustly collaborate in C-0.75. This observation convincingly confirms that the newly-minted 1.5-nm mesopores (double the ion diameter) do underlie a more efficient but distinct energy storage mode, mainly resulting in the capacitance boost. The energy storage efficiency in 1.5 nm pores considerably exceeds that inside the 0.8 nm micropores. Aside from a single ion layer confinement in ion-sized pores measuring 0.8 nm, the 1.5 nm pores ideally double the ion size, and hence almost accommodate two counter-ion layers through more efficient ion packing, not just by sacrificing pore accessibility. In essence, the anomalous capacitance modification must stem from both the highly reinforced degree of confinement and the constructive double electric layer overlapping behavior of the adsorbents inside 1.5 nm pores, by which the detrimental over-screening of the long-range interionic electrostatic correlation networks is totally inhibited to yield a more amplified ion partitioning (featured by a larger capacity). Through consolidating both effects, a stronger electrostatic counteraction (by the carbon pore sidewall) of the Coulombic repulsion interactions between ions of the same charge (namely the stronger net surface charging of the carbon atoms) could also proceed, allowing for “geometrically confined preferential” ion stacking in the carbon nanospaces. Specifically, the confinement-induced high-energy phase and conformation transition of ILs within the nanocavities occurs, as explored by the previous studies [52,61,62,63,64]. Making full use of the new effects found here, SCs (possessing favorable cost, sustainability, rate, and lifetime traits, despite having the major shortcoming of low specific energy density) may be improved to reach the performance of batteries. Although it appears very tough to predict this prospect, we are sure that both using the smaller or higher coordinating ionic liquids and optimizing the carbon sidewall–ion interplays to facilitate the smooth electrochemical reactions within finely modulated nanopores may enable further bridging of the gulf between SCs and the present battery solutions.

All in all, decoupling their respective contributions in carbon electrode materials featuring varying pore widths and heteroatom functionalization carries great potential for further enhancement of carbon-based energy storage devices. For example, our discovery proves that incorporating the specific adsorption centers toward reinforcing the carbon sidewall–electrolyte ion affinity, and so toward steadying the high energy electrolyte decoration, constitutes a potential strategy for further augmenting the energy–power density ratios of supercapacitors, in contrast to the endless search for ever-greater ultra-micropores and surface areas. Eventually, the unraveled effects may unleash an uncharted potential to optimize the energy storage modes and efficiency in electrolyte-dependent SCs via controllably regulating the electrolyte constituents (for example, through the introduction of trace inorganic salts or solvents, or by changing the ILs’ ion nature or type).

## 5. Conclusions

In summary, salt-templated carbons featuring a progressively evolved pore architecture and surface chemistry are developed from cheap and abundant gelatin biopolymers and used as electrodes to explore the structure–performance relation in an electric double layer capacitor with KOH and EMIBF_4_ as electrolytes. Eco-friendly KNO_3_ salts can intercalate in situ into a biopolymer framework to serve as a template, porogen, or activator, and in doing so tune gelatin’s allosteric process during pyrolysis. The pore size and doping level of such biocarbons are adjusted elaborately by tailoring salt/gelatin ratios to scrutinize the effects of such factors on both ionic diffusion kinetics and charge storage modes in two electrolytes. Although specimens with mixed single- and double-ion-sized pores show the best performance regardless of electrolytes, alien doping is found to be more powerful in triggering pseudocapacitance to boost performance for KOH, while chasing a proper pore size has been proven to be more rewarding for EMIBF_4_. In EMIBF_4_, a monolayer confinement of inclusion of single ions into ion-sized micropores and a bilayer confinement of partitioning of ions into double ion-sized mesopores co-exist and synergize to induce the high-energy electrochemical event. This newly induced electrochemical process is most possibly linked with the molecular rearrangements, phase changes, and coordination numbers into the bulk of electrolytes, in addition to the charge storage in the electric double layers. Benefiting from this superb ion–pore size compatibility, a maximum energy density of 88.3 Wh kg^−1^ at 1 kW kg^−1^ in EMIBF_4_ enters the first echelon of advanced carbon-based SCs. Preliminary insights enabled by our series of samples show that there is a significant contribution to the energy storage mechanism depending on structural parameters and ion natures, providing a promising design criterion for SCs.

## Figures and Tables

**Table 1:** The textural and compositional properties of all samples.

Samples	S_total_ (m^2^ g^−1^) ^a^	S_meso_ (m^2^ g^−1^)	S_micro_ (m^2^ g^−1^) ^b^	V_total_ (cm^3^ g^−1^) ^c^	V_meso_ (cm^3^ g^−1^)	V_micro_ (cm^3^ g^−1^) ^b^	Nitrogen Content (at%) ^d^
C-0.25	1084.3	45.5	1038.8	0.403	0.001	0.402	9.45
C-0.5	2050.3	280.2	1770.1	0.825	0.107	0.718	5.52
C-0.75	2744.6	880.5	1864.1	1.304	0.478	0.826	1.68

^a^ Calculated by multi-point Brunauer–Emmett–Teller (BET) method; ^b^ calculated by t-plot method (the adsorption thickness was controlled at 0.3–0.5 nm); ^c^ calculated from quenched solid density function theory (QSDFT) equilibrium model; ^d^ determined by X-ray photoelectron spectroscopy (XPS) tests. S_total_, S_meso_, and S_micro_ represent the specific surface area contributed by all pores, mesopores and micropores, respectively; V_total_, V_meso_, and V_micro_ mean the pore volume contributed by all pores, mesopores, and micropores, respectively.

**Table 2:** A summary of the key performance parameters, such as the specific capacitance, rate capacity, energy–power density, and contribution of the diffusion-controlled process in both electrolytes.

Samples	C (F g^−1^) at 0.5 A g^−1^	C (F g^−1^) at 10 A g^−1^	Rate Capacity from 0.5 to 10 A g^−1^	Energy Density (Wh kg^−1^)	Energy Density (Wh kg^−1^)	Diffusion-Controlled Process
C-0.25-EMIBF_4_	4.8	1.7	35.0%	2.7 at 1 kW kg^−1^	0.94 at 20 kW kg^−1^	47.3%
C-0.5-EMIBF_4_	54.7	22.4	41.0%	30.4 at 1 kW kg^−1^	12.4 at 20 kW kg^−1^	24.1%
C-0.75-EMIBF_4_	158.9	85.9	54.0%	88.3 at 1 kW kg^−1^	47.7 at 20 kW kg^−1^	16.0%
C-0.25-KOH	180.5	133.2	73.8%	5.2 at 0.1 kW kg^−1^	2.5 at 5 kW kg^−1^	28.5%
C-0.5-KOH	205.8	158.5	77.0%	5.6 at 0.1 kW kg^−1^	3.1 at 5 kW kg^−1^	28.3%
C-0.75-KOH	215.6	182.8	84.79%	6.0 at 0.1 kW kg^−1^	3.8 at 5 kW kg^−1^	27.3%

**Table 3:** Comparison of the energy density of our optimal sample with the counterparts of some hitherto-reported advanced carbon materials. All specific energy densities listed here as references are based upon the mass of the active materials of the cell.

Carbon Type	Energy Density	Ionic Liquids	Specific Capacitance (F/g)	Refs
C-0.75	88.3 Wh/kg at 1 kW/kg	EMIMBF_4_ (4V)	160 F/g at 0.5 A/g	This work
N-doped ginger straw carbon	37.8 Wh/kg at 1kW/kg	EMIMBF_4_ (3V)	122 F/g at 0.5 A/g	[32]
Beehive-like hierarchical nanoporous biocarbon	43.3 Wh/kg at 1kW/kg	EMIM-TFSI (3.5 V)	146 F/g at 0.2 A/g	[21]
Cross-coupled macro-mesoporous carbon network	92 Wh/kg at 1kW/kg	EMIBF_4_ (4 V)	166 F/g at 0.5 A/g	[33]
N-doped carbon nanofibrous microspheres	58.7 Wh/kg at 0.3 kW/kg	EMIM-TFSI (3.5 V)	113 F/g at 5 mV/s	[34]
Diamine/triamine functionalized graphene network	51 Wh/kg at 0.552 kW/kg	BMIMBF_4_ (3.5 V)	119 F/g at 10 mV/s	[35]
O-N-S co-doped 3D hierarchical porous carbons	107 Wh/kg at 0.9 kW/kg	BMIMBF_4_ (3.6 V)	214 F/g at 1 A/g	[12]
Graphene	61 W h kg^−1^ at 1kW/kg	Butyrolactone/ BMIMBF_4_ (3.7 V)	131 F/g at 0.1 F/g	[36]
Nitrogen- and sulfur-enriched porous bio-carbon	77.8 Wh/kg at 0.5 kW/kg	NaClO_4_/EC/DMC (3V)	249 F/g at 0.5 A/g	[37]
Interconnected hemp-derived carbon Nanosheets	50 Wh/kg at 1 kW/kg	BMPY TFS (3 V)	122 F/g at 1A/g	[38]
High-surface area bimodal micro-mesoporous biocarbon	60 Wh/kg at 0.1 kW/kg	EMIM TFSI/AN (3V)	160 F/g at 1 A/g	[39]
Pulp mill sludge-derived porous carbon	51 Wh/kg at 375 W/kg	TEABF_4_/AN (3V)	163 F/g at 0.1 A/g	[40]
CNTs	47 Wh/kg at 1 kW/kg	EMIMBF_4_ (3V)	150 F/g at 5 mV/s	[41]
Graphene-like nitrogen-doped biocarbon nanosheets	51 Wh/kg at 362 W/kg	EMITFSI/EMIBF_4_ (3V)	162 F/g at 0.5 A/g	[42]
High specific surface area hierarchical porous biocarbon microspheres	57 Wh/kg at 60 W/kg	TEABF_4_/AN (2.3 V)	308 F/g at 2 mV/s	[43]
Highly porous graphitic biomass carbon	50.16 Wh/kg at 0.3 kW/kg	EMIM-TFSI (3V)	non	[19]
Biomass-derived activated carbon	24.3 Wh/kg at 1kW/kg	PVA/PVP/EMIHSO_4_/HQ (1.2 V)	485 F/g at 0.85 A/g	[44]
Interconnected porous carbon nanosheets	45.5 Wh/kg at 750 W/kg	EMIMBF_4_ (3V)	147 F/g at 1A/g	[45]
Unusual Interconnected graphitized carbon nanosheets	64.7 Wh/kg at 1kW/kg	EMITFSI/EMIBF_4_ (3.5 V)	152 F/g at 1 A/g	[46]
Mesoporous graphene nanoflakes	32.8 Wh/kg at 0.7 kW/kg	N^+^Et_4_TFSI/CH_3_C (3V)	105 F/g at 0.5 A/g	[47]
Hierarchical porous biocarbons	72.3 Wh/kg at 1.4 kW/kg	PVA/LiCl (0.8 V)	226.0 F/g at 0.5 A/g	[48]
Hierarchical porous carbon	4.2 Wh/kg at 0.1kW/kg	KOH (1V)	171 F/g at 1A/g	[49]
Cu-doped porous carbon from heavy metal-contaminated sewage sludge	7.6 Wh·/kg at 0.3 W/kg	KOH (1.2 V)	159 F/g at 0.5 A/g	[50]
Polypyrrole/carbon fiber	5.87 Wh/kg at 60.0 W/kg	PVA/H_3_PO_4_ (1.2 V)	308.2 F/g at 5 mV/s	[51]
N-doped templated carbons	76 Wh/kg at 1kW/kg	EMImBF_4_ (3.5 V)	178 F/g at 0.2 A/g	[52]
